# Visual outcomes and headache in intracranial hypertension following cerebral venous sinus thrombosis: A retrospective cohort study

**DOI:** 10.1371/journal.pone.0362200

**Published:** 2026-11-06

**Authors:** Theresia Knoche, Ivo Grueninger, Charlotte Pietrock, Zohreh Jami, Leon Alexander Danyel

**Affiliations:** 1 Department of Neurology, Charité – Universitätsmedizin Berlin, Berlin, Germany; 2 Department of Otorhinolaryngology, Helios Hospital Berlin-Buch, Berlin, Germany; 3 Department of Ophthalmology, Charité – Universitätsmedizin Berlin, Berlin, Germany; Government Villupuram Medical College and Hospital, INDIA

## Abstract

Intracranial hypertension is a frequent complication of cerebral venous sinus thrombosis (CVT), posing a risk for permanent visual impairment. However, long-term visual outcomes have not been systematically assessed using objective neuro-ophthalmic measures. We retrospectively evaluated consecutive adults presenting to a single-center tertiary intracranial hypertension clinic between 2019 and 2025 with intracranial hypertension following CVT. Visual outcomes were assessed using best-corrected visual acuity, automated perimetry, and optical coherence tomography measuring global peripapillary retinal nerve fiber layer thickness. Headache phenotype and medication overuse were documented. Nineteen patients (11 female; mean age 33.4 ± 12.1 years) were included, with a mean follow-up of 3.4 ± 2.4 years after diagnosis of CVT-related intracranial hypertension. The transverse sinus was involved in 78.9% of cases, and complete or near-complete recanalization was achieved in 68.4%. Although visual acuity remained normal in most patients (84.2%), 26.3% had severe visual field loss and 15.8% showed retinal nerve fiber layer thinning. Headache persisted in 94.7%, most commonly as tension-type headache (63.2%), and 26.3% fulfilled criteria for medication overuse headache. Despite generally preserved visual acuity, substantial visual field deficits and persistent headache were common in CVT-related intracranial hypertension. These findings support routine neuro-ophthalmic follow-up to facilitate early detection of visual deterioration in CVT-related intracranial hypertension. As this cohort was drawn from a specialized tertiary referral clinic, these findings should be interpreted as hypothesis-generating rather than representative of the broader CVT population.

## Introduction

Cerebral venous sinus thrombosis (CVT) is a cerebrovascular disorder characterized by thrombotic occlusion of the dural venous sinuses or cerebral veins, predominantly affecting young adults and women [1–3]. The incidence of CVT has increased over the past decade, with contemporary estimates ranging from 1.3 to 2.0 per 100,000 person-years in Norwegian and U.S. cohorts [2,3]. Although 80% to 90% of patients achieve functional independence, a substantial proportion experience chronic symptoms – including headache, cognitive impairment, and visual disturbances – that negatively impact quality of life [1,4,5].

Intracranial hypertension is the most frequent clinical manifestation of CVT, reported in 10–85% of patients, and may present as the sole feature in up to 40% of cases [5–7]. Prolonged elevation of intracranial pressure with papilledema can lead to progressive visual field loss, and, if left untreated, blindness [8]. In a multicenter study, 40% of CVT patients with papilledema had residual visual field defects on automated perimetry, and higher papilledema grades were strongly associated with permanent visual impairment [8]. Pediatric data similarly demonstrate that over half of children with CVT-related papilledema develop permanent ophthalmic sequelae [9]. Despite the recognized risk of visual loss, current guidelines recommend close monitoring of visual fields and papilledema severity without specifying objective outcome measures [1,10].

In contrast, the management of idiopathic intracranial hypertension (IIH) routinely incorporates serial automated perimetry, optical coherence tomography (OCT) with measurement of retinal nerve fiber layer (RNFL) thickness, and standardized visual acuity testing [11–13]. OCT-derived RNFL thickness correlates with papilledema severity and cerebrospinal fluid (CSF) opening pressure, and changes in RNFL after treatment are prognostic markers of long-term visual field improvement [11]. Perimetry remains the standard of care for serial monitoring of patients with intracranial hypertension and papilledema [11,13]. While automated perimetry and OCT-derived RNFL thickness have each been applied individually in CVT-related intracranial hypertension [8] no study to date has combined visual acuity, perimetry, and RNFL thickness to characterize long-term visual outcomes in this population [14,15]. Existing data rely predominantly on patient-reported symptoms or basic visual acuity, limiting the ability to quantify visual impairment, identify risk factors for irreversible loss, and compare outcomes with the well-characterized IIH population [6,8].

Headache is the most common symptom of CVT, present in nearly 90% of patients at diagnosis, and persists in 46–59% during long-term follow-up, with tension-type and migraine-like phenotypes predominating [16,17]. Persistent headache is more common among patients presenting with isolated intracranial hypertension and may reflect ongoing elevated intracranial pressure, venous outflow impairment, or secondary headache disorders including medication overuse [10,16]. Despite this high prevalence, systematic characterization of headache phenotype, severity, and medication overuse in patients with CVT-related intracranial hypertension remains limited.

We present a cohort of patients who presented to our intracranial hypertension outpatient service following CVT, with standardized follow-up including headache outcomes, visual acuity, automated perimetry, and OCT-derived RNFL thickness. This study aims to provide one of the first objective, quantitative assessments of visual and headache outcomes in this population.

## Materials and methods

### Study design and population

This single-center, observational retrospective cohort study included all consecutive adult patients (≥18 years) who presented to the specialized intracranial hypertension outpatient clinic at Charité – Universitätsmedizin Berlin between January 2019 and December 2025 for evaluation following a prior diagnosis of CVT. Only patients with a confirmed history of CVT and subsequent intracranial hypertension were considered for inclusion.

### Eligibility criteria

- Inclusion criteria: Eligible patients were required to have been diagnosed with CVT-related intracranial hypertension at least 12 months prior to their first presentation at our clinic to allow assessment of long-term outcomes. In addition, documentation of papilledema and CSF opening pressure ≥25 cmCSF at the time of initial diagnosis was mandatory to confirm the presence of intracranial hypertension. Eligibility was based on documented CVT-related intracranial hypertension at initial diagnosis; ongoing intracranial hypertension at the time of the follow-up evaluation was not required.
- Exclusion criteria: Patients were excluded if intracranial hypertension could not be verified based on available clinical documentation, if they lacked ophthalmological follow-up data, or if alternative causes of intracranial hypertension were identified. Specifically, patients with competing etiologies, such as stent thrombosis and concurrent ophthalmic disease, were excluded. Patients who did not attend the scheduled neuro-ophthalmological examination were considered lost to follow-up and excluded from the final analysis.

### Clinical setting

Since 2019, the Department of Neurology at Charité – Universitätsmedizin Berlin, a tertiary care center, has operated a nationwide specialized outpatient clinic for the management of intracranial hypertension, in close collaboration with the Department of Ophthalmology. Patients are referred by their treating neurologists and are required to provide comprehensive documentation of their medical history, including prior neurological and ophthalmological evaluations, neuroimaging studies, and disease-specific reports at the time of their first visit. All patients undergo a standardized neurological and neuro-ophthalmological evaluation. The ophthalmological assessment is performed on the same day or in the following week and includes measurement of best-corrected visual acuity (BCVA), visual field testing, and OCT. Reported visual and headache outcomes reflect this standardized cross-sectional evaluation at our center, with the term ‘follow-up’ used to denote the interval between CVT diagnosis and the standardized assessment at our center.

### Data collection and management

This study was conducted in accordance with the Declaration of Helsinki and approved by the ethics committee of Charité – Universitätsmedizin Berlin (application no. EA4/004/21). Due to the retrospective nature of the study and the use of anonymized clinical data obtained as part of routine care, the requirement for informed consent was waived. All procedures complied with institutional guidelines and adhered to the Strengthening the Reporting of Observational Studies in Epidemiology (STROBE) statement [18]. The corresponding STROBE checklist is available as Supporting Information (S1 Table).

Data were retrospectively extracted between 1^st^ February and 30^th^ April 2026 from clinical records obtained during routine care. Data were entered and managed in IBM SPSS Statistics (Version 31, 2025); data cleaning involved removing variables not relevant to this analysis and applying the eligibility criteria prior to statistical analyses. Only the corresponding author had access to information that could identify individual participants during data collection. Data were extracted directly from clinical records by the corresponding author and entered into an anonymized table; co-authors did not have access to identifiable source data at any stage. A de-identified minimal dataset underlying the findings of this study is provided as Supporting Information (S2 File). As this study used routinely collected clinical data, no blinding was performed; OCT/perimetry technicians and the corresponding author performing data extraction had access to patients’ clinical history, including prior CVT course and recanalization status.

The following variables were collected:

- Demographic and baseline characteristics: age, sex, body mass index (BMI), CSF opening pressure at CVT diagnosis, disease duration (time between CVT diagnosis and visual follow-up)
- CVT-related variables: underlying etiology, localization of thrombosis, and number of neuroimaging studies (MRI) performed, recanalization status was assessed on follow-up MR venography and classified using a four-grade system: No recanalization, incomplete recanalization, near-complete recanalization, and complete recanalization [19,20].
- Clinical symptoms: presence and characteristics of headache (including intensity measured by numeric rating scale [NRS], phenotype [assessed via structured interview at the time of presentation to the specialized outpatient clinic by a specialist in neurology, according to International Classification of Headache Disorders, 3rd edition (ICHD-3) criteria], and medication overuse), as well as associated symptoms such as pulsatile tinnitus, vertigo, diplopia, and visual disturbances (blurred vision and transient obscuration)
- Treatment strategies: medical and surgical interventions for intracranial hypertension, including pharmacological therapy and CSF diversion procedures (e.g., ventriculoperitoneal (VP) shunting)
- Ophthalmological variables: visual acuity, automated perimetry, dilated fundus examination findings, and RNFL thickness measured by OCT

### Visual outcome assessment

The primary outcomes of this study were objective visual function parameters. Not all baseline ophthalmologic examinations were performed at our clinic but at referring institutions, using various OCT and perimetry devices; therefore, direct comparison of baseline and follow-up measurements was not performed due to non-interchangeability of measurements between devices. However, follow-up examinations were standardized at our institution. Visual outcomes are reported as final values at last follow-up and as categorical classifications.

BCVA of the most severely affected eye was transformed to logMAR (logarithm of the minimum angle of resolution) for statistical analysis (logMAR = −log (decimal visual acuity)). Visual acuity was classified according to World Health Organization criteria, as normal (logMAR ≤ 0.30, decimal ≥0.5), mild to moderate visual impairment (logMAR > 0.30 to ≤1.0, decimal 0.5 to 0.1) and severe visual impairment/blindness (logMAR > 1.0, decimal < 0.1). Visual field testing was conducted using automated perimetry (Octopus 900 perimeter, Haag-Streit), applying a 30−2° testing strategy. Only visual fields with good reliability were included (≤1/3 false negatives, ≤ 15% false positives). Visual field loss severity was classified based on mean deviation (MD) using the modified Hodapp-Parrish-Anderson criteria as a pragmatic, standardized severity scale (normal: MD ≥−2 dB, mild visual field loss: MD >−6 dB to −2 dB, moderate visual field loss: MD −6 dB to −12 dB, severe visual field loss: MD <−12 dB) [21]. Average global peripapillary RNFL thickness was measured using spectral-domain OCT (Heidelberg Spectralis™). OCT scans were required to meet the device’s signal-strength quality threshold, with adequate centration and without segmentation failure or artifacts affecting RNFL measurements. Global RNFL thickness values are reported descriptively. For reference, normal mean global RNFL is approximately 97–100 μm, with values declining approximately 1.5 μm per decade of age. RNFL was classified as normal (≥5th percentile), borderline (1st–5th percentile), or abnormal (<1st percentile) based on the device’s normative database.

For per-patient analyses, the worse eye was determined separately for each visual parameter (i.e., the eye with the lower RNFL, lower visual acuity, or more negative MD), rather than a single composite worse eye per patient. This approach was used to capture the maximum severity of each parameter.

### Statistical analysis

Statistical analyses were performed with IBM SPSS Statistics software (Version 31, 2025). Categorical variables were expressed in frequencies and percentages, continuous normally distributed variables as mean ± standard deviation and non-parametric variables as median with interquartile range (IQR). Q-Q plots and histograms were used to check for normal distribution. Missing data were handled by listwise deletion and reporting of valid percentages. Given the small sample size, the absence of a comparator cohort, and the non-interchangeability of OCT and perimetry devices used for baseline assessments at referring institutions, inferential statistical comparisons were not considered methodologically appropriate and were therefore not performed; results are reported descriptively without correction for multiple comparisons. 95% confidence intervals were calculated using the Wilson score method for binomial proportions or the t-distribution for continuous variables.

## Results

### Study cohort

From 2019 to 2025, 24 consecutive adult patients were referred to our outpatient clinic for consultation of pre-diagnosed intracranial hypertension following CVT. Five patients were excluded from further analysis. Fig 1 depicts the patient selection process. The study cohort comprised 19 (11 female, 33.4 ± 12.1 years) patients. Mean BMI was 27.5 ± 9.7 kg/m^2^. Mean CSF opening pressure was 40.0 ± 9.6 cmCSF (Table 1).

**Table 1:** Summary of patient cohort characteristics, including symptom frequencies.

	n = 19 (11 female, 8 male)
**age (years)**	33.4 ± 12.1
**BMI (kg/m**^**2**^)	27.5 ± 9.7
**CSF opening pressure (cmCSF)**	40.0 ± 9.6
**therapy for intracranial hypertension**	
• pharmacological, n • acetazolamide • topiramate	17 (89.5%) 15 (78.9%) 4 (21.1%)
• shunting surgery, n	3 (15.8%)
**symptoms (at follow-up), n**	
• headache	18 (94.7%)
• visual symptoms	13 (68.4%)
• vertigo	5 (26.3%)
• pulsatile tinnitus	3 (15.8%)
• diplopia	2 (10.5%)

**Fig 1:**
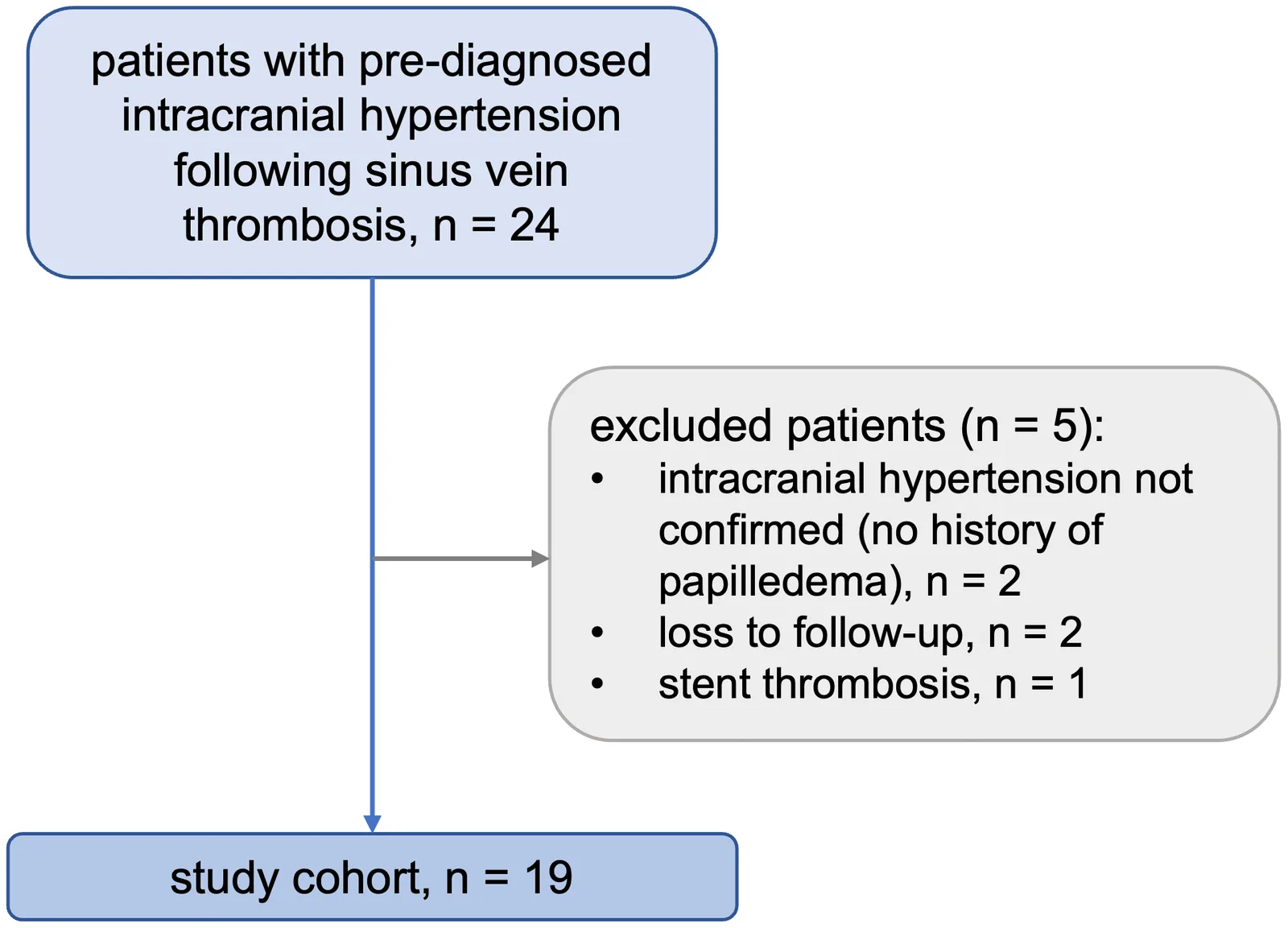
Flow-chart of the patient selection process. A total of 24 patients referred to our outpatient clinic for consultation regarding pre-diagnosed intracranial hypertension following cerebral venous sinus thrombosis (CVT) were considered for study inclusion. Five patients were excluded: intracranial hypertension could not be confirmed in two cases (no history of papilledema), one patient with CVT secondary to stent thrombosis and two because the standardized assessment at our center was not completed.

The most common reason for consultation was treatment for chronic headache in 63.2% (12/19) of patients. Counselling regarding further medication management accounted for 21.1% (4/19) of referrals. Two patients presented regarding shunt-related complications (10.5%, 2/19) and ophthalmological deterioration leading to the diagnosis of a dural arteriovenous fistula in 5.3% (1/19).

The most frequent symptom was headache, present in 94.7% (18/19) of patients, followed by visual symptoms (blurred vision, transient obscuration), vertigo, pulsatile tinnitus and diplopia. Symptom frequencies are presented in Table 1.

Regarding headache characteristics, tension-type headache was the predominant phenotype, observed in 63.2% (12/19) of patients, followed by migraine in 21.1% (4/19). Headache was classified as unspecified in 10.5% (2/19), while 5.3% (1/19) of patients reported no headache at the time of evaluation. Median headache intensity on the numeric rating scale was 4 (Q1-Q3: 3–7.25). International Classification of Headache Disorders (ICHD-3) criteria for medication overuse headache were met in 5 patients (26.3%).

### CVT location & etiology

Overall, the transverse sinus was the most frequently involved site, affected in 78.9% (15/19) of patients, followed by the sigmoid sinus in 68.4% (13/19) and the superior sagittal sinus in 36.8% (7/19, Fig 2). Involvement of the internal jugular vein was observed in 21.1% (4/19) of cases. Less commonly affected structures included the confluens of sinuses (10.5%, 2/19), the sinus rectus (5.3%, 1/19), and cortical/bridging veins (10.5%, 2/19). Multisite involvement was common, with 78.9% (15/19) of patients presenting with thrombosis affecting more than one venous segment (Fig 2).

**Fig 2:**
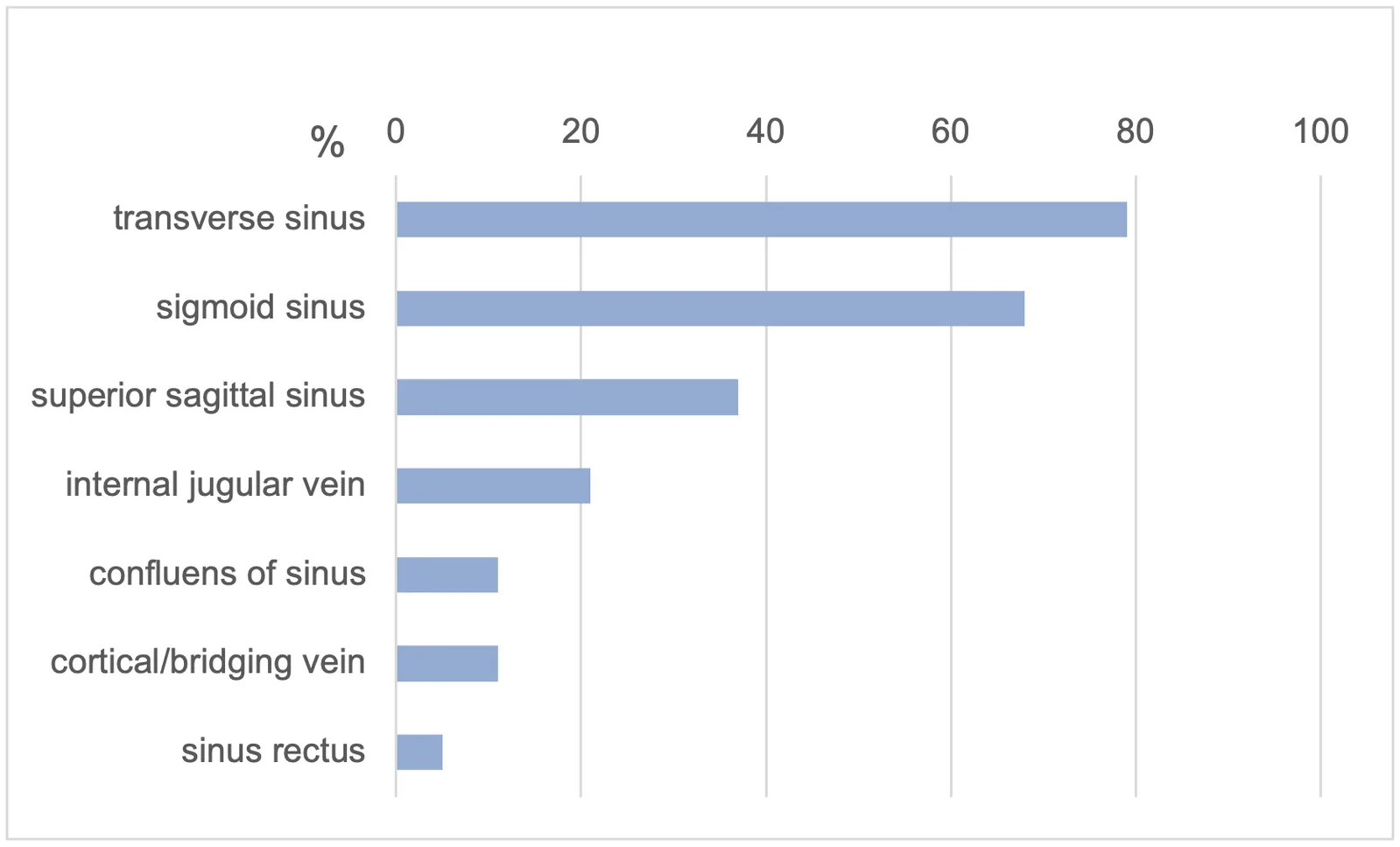
Distribution of venous sinus involvement in CVT patients (n = 19). In 78.9% multiple sites were involved.

Regarding CVT etiology, an identifiable risk factor was present in 63.2% (12/19) of patients. This comprised prothrombotic conditions in 47.4% (9/19) including thrombocytosis/polycythemia vera (15.8%, 3/19), Factor-V-Leiden mutation/APC resistance (15.8%, 3/19), prothrombin mutation (5.3%, 1/19), elevated factor VIII levels (5.3%, 1/19), and antiphospholipid syndrome (5.3%, 1/19) – together with septic CVT secondary to bacterial infections of the middle ear or paranasal sinuses in 10.5% (2/19), and glucocorticoid-associated CVT in 5.3% (1/19). CVT etiology remained unclear in 36.8% (7/19) of patients; oral contraceptives were discussed as a contributing factor in 2 of these cases.

### Treatment

All patients received anticoagulation therapy following CVT diagnosis, with the choice of anticoagulant guided by the underlying etiology.

For the treatment of intracranial hypertension, the majority of patients received pharmacological therapy, administered in 89.5% (17/19) of cases. At the time of consultation, treatment of intracranial hypertension had been completed in 58.8% (10/17) and was ongoing in 41.2% (7/17) of these patients. The most common medication was acetazolamide, prescribed in 78.9% (15/19) of patients, with a mean daily dose of 975 ± 468 mg. Topiramate was used less frequently, in 21.1% (4/19) of patients, with a mean daily dose of 112.5 ± 75 mg (Table 1). Two patients received a combination of acetazolamide and topiramate. No patient received furosemide. Surgical treatment with a ventriculoperitoneal (VP) shunt was performed in 15.8% (3/19) of patients.

Patients underwent repeated neuroimaging, with a median of 4 (IQR 6) MRIs conducted since the initial diagnosis of CVT.

Recanalization status was assessed on follow-up MR venography and classified using a four-grade system: No recanalization, incomplete recanalization, near-complete recanalization, and complete recanalization [20,22]. Complete recanalization was observed in 52.6% (10/19) of patients, near-complete recanalization in 15.8% (3/19), and incomplete recanalization in 26.3% (5/19). One patient (5.3%) had no recanalization. Overall, 94.7% (18/19) of patients achieved some degree of recanalization, with 68.4% (13/19) achieving complete or near-complete recanalization.

### Visual outcome

Visual outcomes were assessed at a mean follow-up of 3.4 ± 2.4 years after diagnosis. Mean visual acuity at follow-up was 0.08 ± 0.15 logMAR (n = 18; one patient with a decimal acuity of 0 was excluded, as logMAR is undefined for this value) and 0.82 ± 0.29 decimal (n = 19). The majority of patients (84%, 16/19) had normal visual acuity (logMAR ≤ 0.30), while 10.5% (2/19) had mild to moderate visual impairment and 5.3% (1/19) had severe visual impairment or blindness.

Mean visual field MD was −7.7 ± 8.1 dB, indicating moderate visual field loss on average. Visual field outcomes were heterogeneous: 36.8% (7/19) had normal visual fields (MD ≥−2 dB), 26.3% (n = 5) had mild visual field loss, 10.5% (2/19) had moderate loss, and 26.3% (5/19) had severe visual field loss (MD <−12 dB). Thus, 63.2% (12/19) demonstrated some degree of visual field impairment at follow-up. Mean global RNFL thickness was 97 ± 23 μm. Based on device-specific normative percentiles, 73.7% (14/19) had normal RNFL thickness, 10.5% (2/19) had borderline thinning, and 15.8% (3/19) had abnormal RNFL thinning. Optic disc atrophy was present in 21.1% (4/19) of patients.

Baseline RNFL measurements were available in 13 of 19 patients (68.4%). Mean baseline RNFL thickness was 237 ± 89μm, reflecting papilledema-related swelling at the time of CVT diagnosis. At follow-up, mean RNFL thickness was 97 ± 22 μm (Fig 3). Direct statistical comparison between timepoints was not performed due to methodological heterogeneity, as baseline measurements were obtained on different OCT devices at referring institutions.

**Fig 3:**
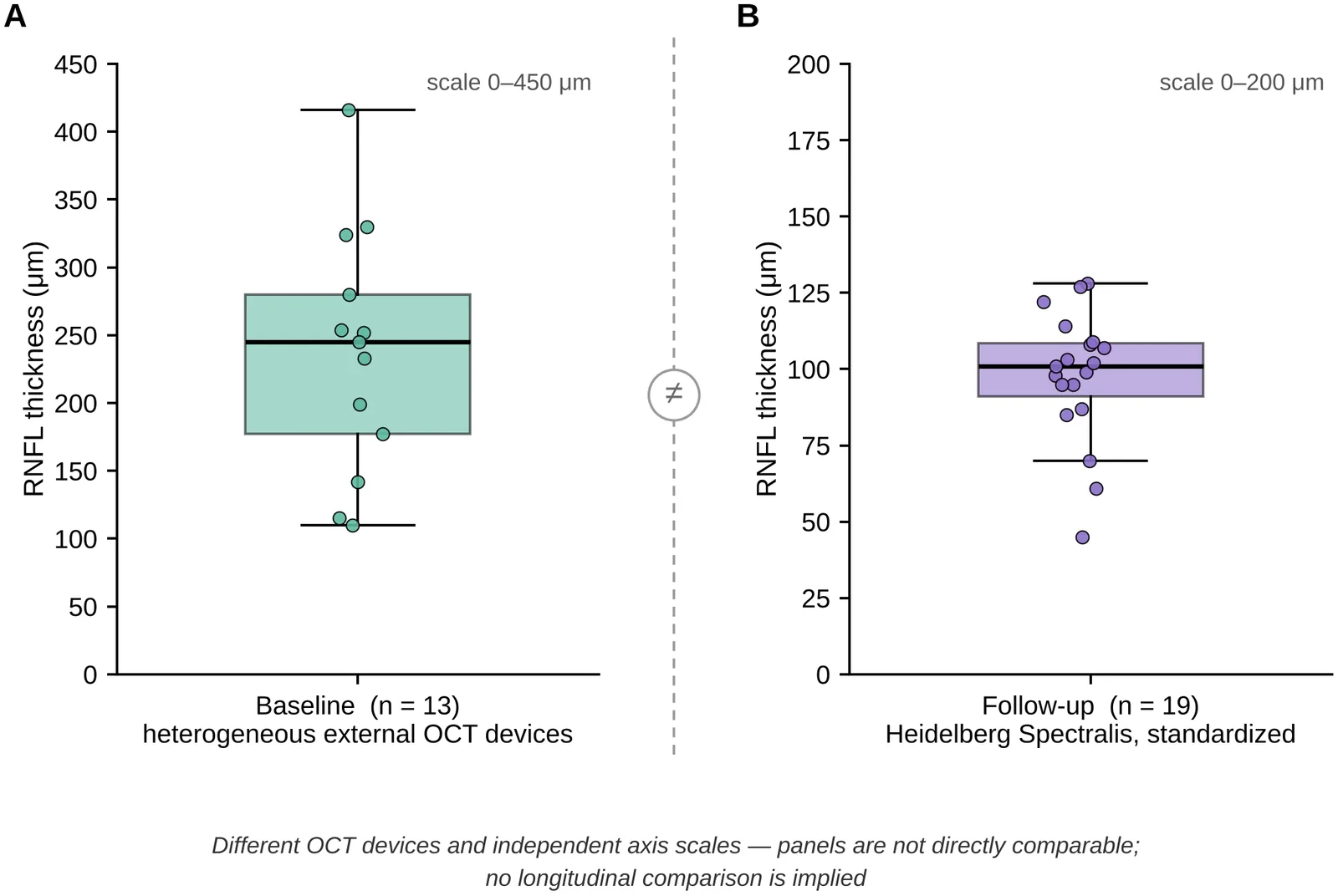
Global peripapillary RNFL thickness at baseline (A) and follow-up (B). Baseline RNFL data were available for 13 of 19 patients (68.4%), whereas follow-up data were available for all 19 patients. The horizontal line within each box represents the median, box edges represent the interquartile range (IQR), and whiskers extend to 1.5 × IQR; individual patient values are overlaid as points. Panels use independent y-axis scales and are not plotted on a common axis. Because baseline and follow-up measurements were acquired using different OCT devices, values are not directly comparable. The data are presented descriptively and should not be interpreted as evidence of longitudinal change, treatment response, or recovery.

While the majority of patients maintained normal visual acuity (84.2%, 16/19) and normal RNFL thickness (73.7%, 14/19), visual field deficits were common, with 63.2% demonstrating some degree of perimetric abnormality and 26.3% (5/19) having severe visual field loss.

As a sensitivity test, we compared follow-up visual parameters between patients with completed versus ongoing IH-directed pharmacological therapy (n = 10 vs. n = 7). Mean follow-up RNFL was 104.5 ± 18.8 μm in the completed group versus 91.7 ± 21.7 μm in the ongoing group; mean MD was −11.3 ± 8.5 dB versus −7.0 ± 10.5 dB, respectively; visual acuity was similar between groups (0.87 ± 0.20 vs. 0.80 ± 0.37). Key visual examination parameters and distribution of visual outcomes are summarized in Table 2 and 3.

**Table 2:** Descriptive analysis of key visual examination parameters recorded at follow-up (mean values and standard deviation). Decimal visual acuity measurements were converted to logMAR for standardization. Visual field recorded in decibels (dB). Retinal nerve fiber layer (RNFL) was assessed using optical coherence tomography (OCT). All 19 patients had visual acuity, perimetry, and RNFL at the final assessment. One patient with a decimal visual acuity of 0 is excluded from the logMAR analysis (n = 18), as logMAR is undefined for this value; this patient is included in the decimal and categorical analyses.

	n = 19	95% CI
**visual acuity (logMAR)**	0.08 ± 0.15*	0.01–0.15
**visual acuity (decimal)**	0.82 ± 0.29	0.68–0.96
**visual field mean deviation (dB)**	−7.74 ± 8.1	−11.64 – (−3.84)
**RNFL (μm)**	97 ± 22.5	86.2 −107.8
**optic disc atrophy, n**	4/19 (21.1%)	8.5–43.3%
**disease duration (years)**	3.4 ± 2.4	2.24–4.56

**Table 3:** Distribution of visual outcomes across severity categories. Visual acuity according to World Health Organization criteria as normal (logMAR ≤ 0.30, decimal ≥0.5), mild to moderate visual impairment (logMAR > 0.30 to ≤1.0, decimal 0.5 to 0.1) and severe visual impairment/blindness (logMAR > 1.0, decimal <0.1). Visual field loss severity was classified based on mean deviation (MD) (normal: MD ≥−2 dB, mild visual field loss: MD >−6 dB to −2 dB, moderate visual field loss: MD −6 dB to −12 dB, severe visual field loss: MD <−12 dB) [21]. Global peripapillary retinal nerve fiber layer (RNFL) thickness was classified as normal (≥5th percentile), borderline (1st–5th percentile), or abnormal (<1st percentile) based on the device’s (Heidelberg Spectralis™) normative database.

outcome parameter	category	n = 19	95% CI
**visual acuity**	normal	16 (84.2%)	62.4–94.5%
	mild – moderate impairment	2 (10.5%)	2.9–31.4%
	severe impairment / blindness	1 (5.3%)	0.9–24.6%
**visual field loss**	normal	7 (36.8%)	19.1–59.0%
	mild	5 (26.3%)	11.8–48.8%
	moderate	2 (10.5%)	2.9–31.4%
	severe	5 (26.3%)	11.8–48.8%
**global RNFL**	normal	14 (73.7%)	51.2–88.2%
	borderline	2 (10.5%)	2.9–31.4%
	abnormal	3 (15.8%)	5.5–37.6%

## Discussion

This study provides one of the first systematic assessments of long-term visual outcomes using objective measures – visual acuity, perimetry, and OCT – in patients with intracranial hypertension following cerebral venous thrombosis. At a mean follow-up of 3.4 years, we observed a notable dissociation between preserved visual acuity (84.2% normal) and frequent visual field impairment (63.2% abnormal), a pattern consistent with the peripheral-to-central progression of papilledema-related optic neuropathy [13]. Although objective visual measures have previously been applied in CVT-related intracranial hypertension, prior studies used a single modality or shorter follow-up. A retrospective cohort of 65 patients with CVT-associated papilledema reported residual visual field defects in 40% of patients, with higher papilledema grade predicting permanent field loss, but provided no OCT/RNFL assessment [8]. Other studies have objectively characterized visual field impairment and papilledema in relation to CSF opening pressure, but likewise did not include OCT [14]. Conversely, OCT-based studies in CVT have been prospective but short-term, assessing RNFL after 2-month follow-up without a visual field or acuity outcome [15]. Large CVT outcome datasets report residual visual impairment using composite, clinician-assigned categories rather than quantitative perimetric or OCT measures [6]. To our knowledge, the present study is the first to combine standardized visual acuity, perimetric mean deviation, and OCT-derived RNFL thickness to describe visual outcomes in this population. Descriptively, the frequency of visual field impairment in our cohort appears at least as high as that reported in IIH cohorts, where visual field abnormalities are detected in 87–92% of patients at onset, with favorable outcomes (MD ≥−4 dB) achieved in approximately 75–90% after treatment [23,24]; however, in the absence of a matched IIH group in the present study, this comparison should be regarded as descriptive. Additionally, this study did not capture baseline papilledema severity, a known predictor of visual outcome in IIH; without this variable, we cannot distinguish whether CVT-associated intracranial hypertension carries an intrinsically worse visual prognosis than IIH, or whether this cohort simply presented with more severe papilledema at baseline. Only 36.8% of our patients had normal visual fields at follow-up, and 26.3% (95% CI: 11.8–48.8%) had severe visual field loss (MD <−12 dB, Table 3). This higher rate of visual field impairment may reflect several factors unique to CVT-related intracranial hypertension: the acute nature of the initial insult, potential delays in diagnosis and treatment of intracranial hypertension while managing the thrombosis itself, and the possibility that venous outflow obstruction from incomplete recanalization contributes to persistent or recurrent intracranial hypertension.

The rate of optic disc atrophy in our cohort (21.1%) is consistent with the known progression from chronic papilledema to optic atrophy. Importantly, RNFL thinning can occur even in patients with apparently effective treatment and resolution of papilledema, representing subclinical axonal loss [25]. Our finding that 15.8% (3/19) of patients had abnormal RNFL thickness supports this observation.

A key finding is that visual acuity remained normal in 84.2% of patients despite frequent visual field deficits. To confirm this dissociation was not an artifact of our eye-selection method, we performed a sensitivity analysis using a single, fixed worse eye per patient (defined by perimetric MD) to reassess visual acuity, rather than selecting each parameter independently. This approach agreed with our original findings in 89.5% (17/19) of patients, with an identical categorical distribution, confirming that the visual acuity/visual field dissociation is not primarily driven by independent per-parameter eye selection. This pattern is characteristic of papilledema-related optic neuropathy, where vision loss progresses from the periphery inward, sparing central acuity until late in the course of disease. Recent work has identified two distinct mechanisms of visual impairment in intracranial hypertension: (1) RNFL and ganglion cell layer atrophy causing peripheral scotomata without affecting acuity, and (2) macular involvement (edema, exudates, or disorganization of inner retinal layers) causing acuity loss, typically only with severe papilledema [26]. Our data suggest that most patients experienced the former mechanism. This dissociation has important clinical implications. Patients may report “good vision” based on preserved central acuity while harboring significant peripheral field loss that affects daily activities such as driving. This underscores the necessity of formal perimetry in the follow-up, rather than relying on visual acuity alone.

Headache was present in 94.7% of our cohort at follow-up, with tension-type headache being the predominant phenotype. As 63.2% (12/19) of patients were referred to our clinic specifically for headache management, this figure should not be interpreted as the prevalence of headache among all CVT survivors or all patients with CVT-related intracranial hypertension but reflects the burden within a headache-enriched referral population. However, this high prevalence is consistent with the literature on post-CVT headache, which affects 46–59% of CVT survivors [16,17]. Notably, isolated intracranial hypertension has been identified as an independent risk factor for severe residual headache after CVT (OR 3.3) [19]

The predominance of tension-type headache phenotype in our cohort is consistent with prior studies showing that tension-type-like headache is the most common post-CVT headache phenotype, observed in approximately 35–53% of patients with post-CVT headache [16,17]. The mechanisms underlying chronic headache in CVT likely include persistent intracranial pressure elevation, incomplete venous recanalization, and central sensitization.

The recanalization rate in our cohort is consistent with pooled estimates from the literature, where approximately 85% of CVT patients achieve recanalization with anticoagulation therapy [20]. The rate of complete recanalization (52.6%) in our cohort is comparable to prior studies reporting complete recanalization rates of 48–68% [22,27]. Despite the high rate of recanalization, a substantial proportion of patients demonstrated persistent visual field deficits and chronic headache. Given the descriptive nature of this study, we cannot determine whether recanalization status is associated with visual or headache outcomes; this would require a dedicated cross-tabulation and adequately powered analysis in future work.

Our findings have implications for clinical practice. First, patients with CVT-related intracranial hypertension require long-term ophthalmological surveillance with formal perimetry and OCT, as visual acuity alone is insufficient to detect visual field loss. Second, the high burden of chronic headache in this population warrants dedicated headache management strategies. Third, the substantial rate of visual field impairment despite treatment emphasizes the need for studies examining whether treatment timing or intensity influences long-term visual outcomes in this population.

### Limitations

This study has several limitations that should be considered when interpreting its findings.

First, the retrospective design is subject to inherent limitations, including reliance on the completeness and accuracy of existing clinical documentation. Second, the small sample size limited the statistical power of our analyses; consequently, only descriptive statistics were reported, and no multivariable analysis could be performed to identify independent predictors of visual or headache outcomes, precluding adjustment for potential confounders such as recanalization status, treatment type, or CSF opening pressure. As a sensitivity test, we compared follow-up visual parameters between patients with completed versus ongoing IH-directed pharmacological therapy: Numerically, patients with completed therapy had thinner RNFL and more negative MD than those still on treatment, though visual acuity was similar between groups; given the small subgroup sizes, these differences should not be interpreted causally and may reflect that patients with more severe or refractory disease remain on therapy longer, or other unmeasured confounding. This heterogeneity in treatment status at the time of a single cross-sectional assessment is an inherent limitation of this study design.

Third, our cohort was drawn from a specialized tertiary-referral outpatient clinic, introducing referral bias: patients referred to us likely represent a more symptomatic or treatment-refractory subset of the broader CVT population, which may overestimate the true burden of visual and headache morbidity after CVT. Relatedly, our inclusion criteria required evaluation at least 12 months after CVT diagnosis and attendance at a scheduled follow-up visit; patients who were lost to follow-up, or recovered fully without seeking further care may be systematically underrepresented.

Fourth, baseline visual examinations were not standardized across institutions, precluding direct statistical comparison of visual function over time; only follow-up measurements obtained at our institution could be compared descriptively.

Fifth, headache assessment was limited to phenotypic classification and symptom presence; standardized instruments evaluating headache impact, quality of life, or monthly headache days were not employed. Pre-existing primary headache disorders were not systematically assessed. Outcome data were not adjusted for treatment strategy, therefore conclusions about the effectiveness or timing of therapeutic interventions, and in patients with visual deterioration, underlying contributory factors (e.g., non-adherence, therapy intensity and duration, delayed treatment initiation) could not be determined.

Finally, given the single-center design, small sample size, and selected referral population, the external validity of our findings to the broader population of patients with CVT-related intracranial hypertension is limited, and our results should be considered hypothesis-generating.

## Conclusions

This study represents one of the first with comprehensive assessment of visual function in patients with intracranial hypertension following CVT. Our findings demonstrate clinically relevant visual morbidity in this selected referral cohort, with severe visual field loss observed in 26.3% (95% CI 11.8–48.8%) of patients. The high prevalence of chronic headache further contributes to disease burden. These findings highlight the need for standardized, objective visual monitoring in this population.

## Supporting information

S1 Table STROBE Checklist. (DOCX)

S2 File Anonymized study dataset. (XLSX)
