# Supplementary material for: Visual outcomes and headache in intracranial hypertension following cerebral venous sinus thrombosis: A retrospective cohort study
Source: PLoS One. 2026 Nov 6;21(11):e0362200. doi: 10.1371/journal.pone.0362200 (PMC13742918; doi:10.1371/journal.pone.0362200)
Supplement: Supplementary file 1 [file pone.0362200.s001.docx]

# **STROBE Statement**

**Study design:** Cohort study

**Manuscript:** "Visual outcomes and headache in intracranial hypertension following cerebral venous sinus thrombosis: a retrospective cohort study"

| **Item** | **STROBE item** | **Reported in manuscript** |
| --- | --- | --- |
| **Title and abstract** | | |
| 1a | Indicate the study's design with a commonly used term in the title or abstract | Title: "...a retrospective cohort study" |
| 1b | Provide an informative and balanced summary in the abstract | Abstract |
| **Introduction** | | |
| 2 | Background/rationale — explain the scientific background and rationale | Introduction, paras 1–4 |
| 3 | Objectives — state specific objectives, including hypotheses | Introduction, final paragraph ("This study aims to provide...") |
| **Methods** | | |
| 4 | Study design — present key elements early in the paper | Methods, "Study design and population" |
| 5 | Setting — describe setting, locations, relevant dates (recruitment, exposure, follow-up, data collection) | Methods, "Study design and population" and "Clinical setting and assessment protocol" |
| 6a | Participants — eligibility criteria, sources/methods of selection | Methods, "Study design and population" |
| 6b | For matched studies, give matching criteria and number of exposed/unexposed | N/A — not a matched study |
| 7 | Variables — clearly define all outcomes, exposures, predictors, potential confounders, effect modifiers | Methods, "Visual outcome assessment" and "Data collection" |
| 8 | Data sources/measurement — for each variable of interest, give sources of data and details of assessment methods | Methods, "Visual outcome assessment" and "Data collection" |
| 9 | Bias — describe efforts to address potential sources of bias | Limitations |
| 10 | Study size — explain how study size was arrived at | Results, "Study cohort" (consecutive patients over defined study period) |
| 11 | Quantitative variables — explain how quantitative variables were handled; describe groupings | Methods, "Visual outcome assessment" (WHO acuity categories; modified Hodapp-Parrish-Anderson MD categories; RNFL normative percentiles) |
| 12a | Statistical methods — describe all statistical methods, including those to control for confounding | Methods, "Statistical analysis" |
| 12b | Describe methods for subgroup and interaction analyses | Methods, "Statistical analysis" (descriptive subgroup reporting only; no inferential subgroup testing performed) |
| 12c | Explain how missing data were addressed | Methods, "Statistical analysis" (listwise deletion, valid percentages) |
| 12d | If applicable, describe analytical methods taking account of sampling strategy | N/A |
| 12e | Describe any sensitivity analyses | Discussion (worse-eye and treatment-status descriptive sensitivity analyses, added in response to peer review) |
| **Results** | | |
| 13a | Participants — report numbers at each stage of the study | Results, "Study cohort"; Figure 1 (flow chart) |
| 13b | Give reasons for non-participation at each stage | Figure 1 legend (reasons for exclusion, n=5) |
| 13c | Consider use of a flow diagram | Figure 1 |
| 14a | Descriptive data — characteristics of study participants and exposures | Table 1; Results, "Study cohort" |
| 14b | Indicate number of participants with missing data for each variable of interest | Methods/Results — noted per-variable (e.g., baseline RNFL available in 13/19) |
| 14c | Summarize follow-up time | Results, "Visual outcome" (mean 3.4 ± 2.4 years) |
| 15 | Outcome data — report numbers of outcome events or summary measures | Table 2, Table 3, Results |
| 16a | Main results — give unadjusted estimates and, if applicable, confounder-adjusted estimates and their precision (e.g., 95% CI) | Table 2, Table 3 (95% CIs added in response to peer review); no confounder-adjusted estimates reported (descriptive study, see Statistical analysis) |
| 16b | Report category boundaries when continuous variables were categorized | Methods, "Visual outcome assessment" (WHO/HPA/RNFL percentile cutoffs) |
| 16c | If relevant, translate relative risk into absolute risk for a meaningful time period | N/A — no relative risk estimates calculated |
| 17 | Other analyses — report other analyses done (e.g., subgroup, sensitivity) | Discussion (worse-eye and treatment-status sensitivity analyses) |
| **Discussion** | | |
| 18 | Key results — summarize key results with reference to study objectives | Discussion, opening paragraph |
| 19 | Limitations — discuss limitations, sources of bias/imprecision, direction/magnitude of any bias | Limitations |
| 20 | Interpretation — cautious overall interpretation considering objectives, limitations, multiplicity, similar studies | Discussion; Statistical analysis (explicit descriptive/hypothesis-generating statement) |
| 21 | Generalizability — discuss the external validity of the study results | Limitations (referral bias, tertiary-referral population, external validity) |
| **Other information** | | |
| 22 | Funding — give source of funding and role of funders | Acknowledgements |
